# Clinical decision making when cytology indicates a Warthin tumor

**DOI:** 10.1038/s41598-024-58892-0

**Published:** 2024-04-17

**Authors:** Minna Sirviö, Katri Aro, Mira Naukkarinen, Antti Mäkitie, Jussi Tarkkanen, Jetta Kelppe, Timo Atula

**Affiliations:** 1 Department of Otorhinolaryngology – Head and Neck Surgery, Helsinki University Hospital and University of Helsinki, Kasarmikatu 11-13, FI-00029 Helsinki, Finland; 2 Pathology, Helsinki University Hospital Diagnostic Center, Helsinki, Finland; 3 Division of Ear, Nose and Throat Diseases, Department of Clinical Sciences, Intervention and Technology, Karolinska Institutet and Karolinska University Hospital, Stockholm, Sweden; 4 Research Program in Systems Oncology, Faculty of Medicine, University of Helsinki, Helsinki, Finland

**Keywords:** Salivary gland diseases, Diagnosis

## Abstract

Warthin tumor (WT) is a benign tumor usually affecting the parotid gland. The main diagnostic tool remains ultrasound combined with fine-needle aspiration cytology (FNAC). This study aims to examine how reliably FNAC indicates WT for clinical decision making regarding surgical versus conservative management. We included all patients who underwent FNAC from a parotid gland lesion between 2016 and 2018 at our institution, and whose FNAC revealed WT suspicion. The FNACs were divided into three groups based on the cytology report: certain, likely, and possible WT. The patients were divided into two groups based on having had either surgery or follow-up. We sent a questionnaire to patients who had not undergone surgery in order to obtain follow-up for a minimum of four years. Altogether, 135 FNAC samples, from 133 tumors and 125 patients, showed signs of WT. Of the 125 patients, 44 (35%) underwent surgery, and 81 (65%) were managed conservatively. Preoperative misdiagnosis in FNAC occurred in three (7%) surgically treated tumors. Their FNACs were reported as possible WTs, but histopathology revealed another benign lesion. In the conservatively treated group, two patients underwent surgery later during the follow-up. Cytological statements of WT were seldom false, and none were malignant. The majority of the patients were only followed-up and rarely required further treatment. A certain or likely diagnosis of WT in the FNAC report by an experienced head and neck pathologist is highly reliable in selecting patients for conservative surveillance.

## Introduction

Warthin tumor (WT) is the second most common parotid gland tumor, and it constitutes up to 36% (range, 3–36%) of tumors at this location, which is its predilection site^1–6^. WT more often affects men over 50 years of age, and those who are smokers^1,2,7,8^. It is often asymptomatic and can present as multiple and/or bilateral lesions^2,8^.

Ultrasound combined with fine-needle aspiration cytology (FNAC) is the most common tool for investigating parotid gland masses. The sensitivity of FNAC in differentiating benign from malignant lesions varies between 64 and 90% and the specificity 86–100%^3,9–13^. FNAC accuracy tends to be more reliable in the diagnostics of the most common benign salivary gland tumors, i.e., pleomorphic adenomas and WTs, which pathologists encounter more frequently than malignant tumors^3^. The diagnostic accuracy of ultrasound guided FNAC for WT ranges from 74 to 100%^9–11,14^, sensitivity 70.4–97.5%, and specificity 94.8–100%^5,10,15–19^. In clinical decision-making, positive predictive value (PPV) of FNAC indicating WT is essential. This has been reported to range from 87.5 to 98.1%^5,10,15–19^. Typical and often diagnostic FNAC findings for WT present lymphoid stroma, bilayered and oncocytic epithelium, and cystic spaces filled with viscous material^4,9,10^. However, the usual pitfalls for all FNAC diagnostics include the quality of the sample and limited experience of the pathologist^9,10,12,14^. For example, the quality may remain poor if the sample has not been obtained from a solid component of the tumor, or if it does not contain enough cells, or due to cell death or infarction^9,10,12,14^.

The treatment for WT has traditionally been surgery, but this is known to carry certain risks, such as transient or permanent facial nerve paralysis, ear lobe numbness, Frey’s syndrome, hematoma or hemorrhagia, fistula, and infection^20^. Risk for malignant transformation of a WT has been reported to be low (0.3–1%), which has led to a hypothesis that a more conservative treatment approach might be acceptable^1,9–11,20^. Furthermore, the diagnostic abilities and accuracy of FNAC have been evolving. We thus hypothesize that if the FNAC diagnosis of WT is regarded as certain enough, and the patient is asymptomatic and/ or has comorbidities that constitute contraindications for surgery, follow-up only can be safely applied.

We reviewed all consecutive FNAC samples from parotid gland lesions during a 3-year period at our tertiary-care hospital with a referral area of 1.6 M inhabitants. We included samples that yielded suspicious or clear findings of WT. The aim of this study was to evaluate how a FNAC indicating WT can be utilized for clinical decision-making between surgery and conservative surveillance.

## Material and methods

The local research ethics committee approved the study design (HUS 967/2017), and an institutional study permission was granted (§41/2017, updated §45/2022).

For this longitudinal follow-up cohort study, we included all patients who were examined at the Department of Otorhinolaryngology—Head and Neck Surgery, Helsinki University Hospital (Helsinki, Finland) for a parotid gland tumor and with an FNAC sample analysis stating an obvious or possible finding of a WT. We included patients whose FNAC was taken between January 1st, 2016, and December 31st, 2018. The FNAC data were extracted from the university hospital Pathological Archives (Q-Pati). All samples were evaluated by a few experienced head and neck pathologists only.

**Figure 1:**
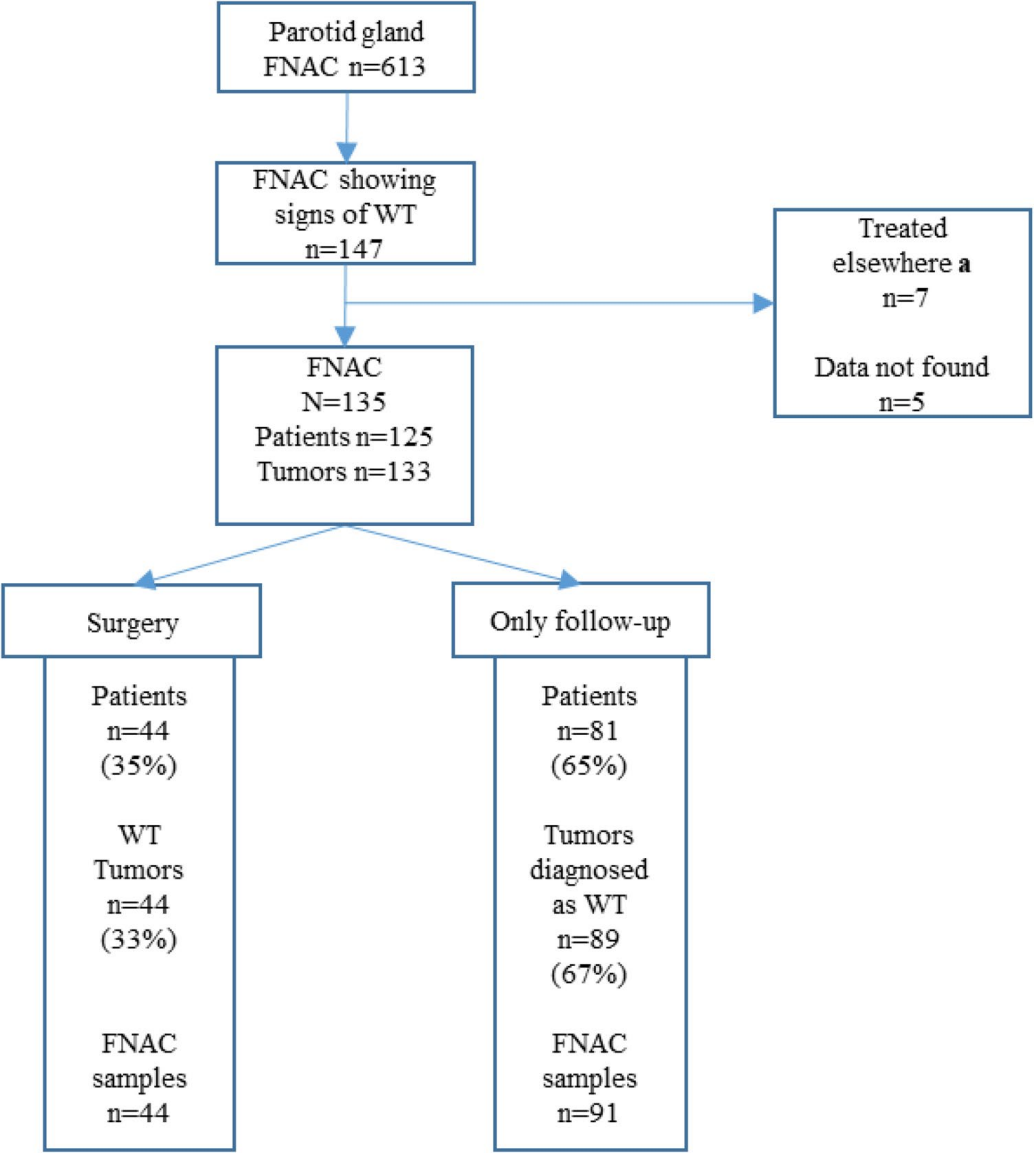
Parotid gland Warthin tumor (WT) FNAC samples, the number of tumors and patients, and their treatment of option (surgery or follow up without surgery) during the years 2016–2018. All in all, the number of patients n = 125, FNAC samples n = 135 and WT tumors n = 133. Surgery and surgically confirmed histology after 2018 are included in the chart. Patients who were not found in the patient data (n = 5) or treated elsewhere (n = 7) were excluded. **a** Patients treated in other Helsinki University hospital departments, e.g., oncology and oro-maxillary surgery.

The data search yielded 613 parotid gland FNAC samples, of which 147 showed signs of WT (Fig. 1). We excluded patients who were managed at other Helsinki University Hospital departments (oro-maxillary surgery, n = 1; dermatology, n = 1; oncology, n = 1; internal medicine, n = 1; primary health care center, n = 3), and those whose clinical data were not available (n = 5). The final study series consisted of 135 FNAC samples, taken from 133 tumors in a series of 125 patients. The patients were divided into two groups: A, patients whose tumors were operated on and thus confirmed histopathologically, and B, those whose tumors were not treated surgically but followed-up only.

We collected data from patient records on clinical patient- and tumor-related factors, treatment, and histology of the surgically treated tumors as well as follow-up information over a minimum of 4 years after diagnosis (until January 2023). In addition, the follow-up data were supplemented with a questionnaire sent in January 2023 for the living patients in the follow-up group (n = 52, 64%), to determine whether any further symptoms related to the salivary glands had developed. Patients were not usually routinely followed-up after the initial visit unless they had new signs or symptoms from the tumor. All patients were advised to contact the hospital if there were any problems after surgery or during follow-up. The follow-up period was calculated from the time of FNAC sample until the date the questionnaire was returned, or the last date when patient records were reviewed, hence giving a maximum follow-up period of January 1st, 2016–January 1st, 2023.

Normality of data were assessed using Kolmogorov–Smirnov test, and visually using histograms. Differences and correlations between parameters and subgroups were assessed using the independent T-test. A *p* value < 0.050 was considered statistically significant.

We divided the WT FNAC reports into three categories: (1) certain (typical findings of WT), (2) likely (high but not certain suspicion of WT), and (3) possible (WT mentioned as one of the tumor alternatives).

### Ethical approval

This study was performed in line with the principles of the Declaration of Helsinki. Approval was granted by the Ethics Committee of the Helsinki University Hospital.

### Consent to participate

Informed consent was obtained from Legally Authorized Representative of the participants.

## Results

The whole cohort comprised 125 patients, 74 (59%) men and 51 (41%) women with a mean age of 67 years (range, 20–92). Fifteen (12%) of them had had an earlier visit at our department due to a WT diagnosis in the same gland before the study cohort period (i.e., before 2016), and 13 (10%) of them had undergone surgery before 2016 because of a prior WT.

Altogether 99 (79%) patients had signs or symptoms of their tumor, the most common being a palpable lump (77%), and 32 (25%) had experienced pain. Twenty-seven (21%) of the tumors were incidental findings, either found by a clinician (n = 5, 19%), or in radiological imaging (n = 22, 81%). The distribution and size of the tumors in the operated and followed-up patient groups are reported in Table 1. The mean tumor size in the operated group was 2.7 cm (median 2.5 cm), and 2.5 cm (median 2.3 cm) in the follow-up group, and the size between groups did not differ significantly (*p* value 0.33).

**Table 1:** Warthin tumor distribution and sizes in the operated and followed up patients.

Patients	Tumor distribution				
	Single unilateral	Multiple unilateral	Single bilateral	Multiple bilateral	Total
Operated	29	8	3	4	44
Followed-up	59	8	7	7	81
Total (n, %)	88 (70)	16 (13)	10 (8)	11 (9)	125 (100)

Patients	Tumor size (cm)				
	Mean	Median	Range	Size reported (n, %)	
Operated	2.7	2.5	1.2–4.6	43 (98)	
Followed-up	2.5	2.3	0.5–5.4	76 (94)	

Regarding tumor management we divided the patients into two groups: 44 (35%) had surgery and 81 (65%) were followed-up only. The mean age between the two groups was 64 years (range, 46–79) in the surgically treated group versus 68 years (range, 20–91) in the follow-up group. No routine follow-up was organized for either group, but patients were able to contact the hospital if they encountered any problems concerning their tumor or surgical site.

In the surgical group, 6 (14%) patients had postoperative adverse events such as infection (n = 3), facial nerve weakness (n = 2), or both (n = 1). Ten (23%) patients had primarily undergone a conservative approach, which was later changed to surgery because of new symptoms such as pain, growth, or infection of the tumor. The patients were not routinely followed-up, but they had contacted the hospital due to their symptoms. The mean time period from diagnosis to surgery among these ten patients was 18.7 months (range, 7–35).

The comparison between FNAC findings and histopathology for the surgically treated group is shown in Table 2. Histological follow up was available in 44/135 cases where FNAC diagnosis was suggestive of WT. In 41/44 cases, typical WT was seen in histology. In the remaining 3/44 cases, one oncocytoma, one lymphoepithelial sialadenitis, and one case suggestive of infarcted WT were found. The one oncotycoma case was re-evaluated according to the latest WHO Classification (2022) of salivary gland tumors^21^. In histological examination, there was a solitary 16 mm well-demarcated encapsulated tumor consisting of benign looking oncocytic cells. Cystic spaces were not seen. No other tumors or diffuse oncocytosis were detected. At the periphery of the tumor there were scattered accumulations of lymphocytes. MAML2-fusion test was not available in our laboratory at the time of the diagnosis to exclude the oncocytic variant of mucoepidermoid carcinoma.

**Table 2:** FNAC samples indicating Warthin tumor.

FNAC diagnosis	FNAC samples (n = 135)	Surgically confirmed tumor histology (n = 44, 33%)		Tumors only followed-up (n = 89, 67%)
Certain Warthin	86	Warthin	26	59
		Necrosis	1^a^	
Likely Warthin	32	Warthin	10	21
Possible Warthin	17	Warthin	5	9
		Oncocytoma	1	
		Lymphoepithelial sialadenitis	1	

The number of patients (n = 125) with parotid gland Warthin tumor (WT), their FNAC samples (n = 135), and tumors (n = 133), and treatment options (surgery or follow-up without surgery) during the years 2016–2018. Surgery and surgically confirmed histology after 2018 are included in the chart. Patients who were not found in the patient data (n = 5) or treated elsewhere (n = 7) were excluded. FNAC is an abbreviation of fine-needle aspiration cytology.

^a^Possible necrotized Warthin tumor.

In the follow-up group, some patients (n = 38, 30%) had occasional out-patient visits or telephone contacts after the first visit, depending on their symptoms. In these cases, the median time from the initial visit to the later out-patient visit, or telephone contact was 1 month (mean 4.7; range, 1–35). At the end of follow-up (until January 1st, 2023), two patients had undergone surgery 1.5–2 years after the initial visit because of tumor growth and pain. In both, the histopathological diagnosis was confirmed to be WT. Also, at the end of the evaluation period, one patient was still waiting for an out-patient visit because of tumor growth. Twenty-nine (36%) patients in this group died of other causes during the evaluation period.

The questionnaire was sent to all the non-surgically treated patients who were alive (n = 52) in the follow-up group and was responded by 25 (48%) patients. This revealed only one patient with more tumor-related symptoms such as significant growth, which led to an experimental sclerotherapy treatment after the study period. Furthermore, 65% (34/52) experienced their WT having either decreased in size or with no change.

## Discussion

We reviewed a series of 125 patients with 135 consecutive parotid gland FNACs referring to a WT over a 3-year period. The primary aim was to evaluate whether surgery can be safely omitted when FNAC indicates signs of WT. We thus investigated the long-term follow-up of the patients managed with surveillance only. The main results indicate that, in our series, where cytological evaluation is centralized to a few head and neck pathologists, certain or likely diagnosis of WT in the FNAC report is highly reliable for selecting patients for conservative follow-up. Of note, due to the present study setting, the exact histopathological diagnosis in the follow-up group could not be definitely determined. Given such a study setting, the aim was not to assess the sensitivity and specificity of FNAC for WT as that can be adequately performed from histologically confirmed series only, and furthermore, these values have been evaluated in other studies.

Although surgery has been the standard recommended treatment for WTs, most of our patients were followed-up only. Further, in the literature, almost all studies on WT and FNAC have focused on the surgically treated patients^1–18,20,22–25^, which is the major difference compared with the present series. Since reporting on cytological findings includes uncertainty in clinical practice, we analyzed the results separately according to various cytological statements (certain, likely, and possible WT). Such a degree of certainty has rarely been addressed in earlier studies^1–18,20,22–26^. Only one third (n = 44) of our patients had surgery and nearly all (n = 41, 93%) preoperative FNAC samples showing signs of a WT turned out to be WT in histopathology as well. Only three (7%) patients had a false positive FNAC diagnosis. These three cytological samples were primarily reported as possible WTs. In these cases, the final histology (oncocytoma, lymphoepithelial sialadenitis, and necrosis) had overlapping histologic features with WT, which may partly explain the discrepancy: oncocytic epithelium—oncocytoma, lymphatic tissue—lymphoepithelial sialadenitis, and necrosis—necrotic changes in an infarcted WT. Previous studies have reported FNAC accuracy for a WT ranging between 51 and 100%^4,10,11,15^. High FNAC accuracy in the present study might at least partly be explained by our FNAC samples having been examined only by a few experienced head and neck pathologists. Similarly to some studies^10,11^, our series revealed no malignancies in the surgical group. However, some have reported malignancies among tumors that have been regarded as WT in FNAC, with the malignancy rate varying from 0.6 to 4.2%^2,3,10,11,14,16,22^. A multicenter study consisting of 483 FNAC samples diagnosed as a WT reported a divergence rate between the FNAC and histological diagnoses ranging from 5.6 to 17.9%^22^, which is slightly higher than in our study. The misdiagnosed FNAC WTs in other studies were often histologically diagnosed as pleomorphic adenoma, oncocytoma, oncocytic cystadenoma, granuloma, mucoepidermoid carcinoma, and acinus cell carcinoma^2,3,10,11,14,22^. The Milan system for staging salivary gland FNACs was introduced in 2018 and was implemented at our clinic in 2019^27–29^. We can speculate that some of the uncertain or possible WT FNACs might have been classified into category III, atypia of undetermined significance (AUS), in the Milan system. This was also seen in the study by Lee et al. where WT was a common histological result in the AUS category^27,28^. The more certain WT FNACs in our series would have most likely been placed into category IV, benign neoplasia.

The histological examination in the present series revealed one possible necrotized WT, for which a preoperative FNAC had been reported as a certain WT. Similar findings have also been reported by others: Sood et al.^14^ found one infarcted and two metaplastic WTs out of 36 cases (8%), and Viguer et al.^16^ one necrotized WT out of 116 cases (1%). This phenomenon is thought to be linked to the features of WT, namely being thin walled and poorly circulated, and thus being sensitive to trauma, e.g., aspiration cytology, that could lead to infarction or inflammation. Histopathological diagnosis after a FNAC can sometimes be difficult because of the necrosis^23^. The infarcted WT can also be considered metaplastic, as it can have squamous epithelial metaplasia. Furthermore, the metaplastic/ infarcted tumor can sometimes be difficult to differentiate from a low-grade mucoepidermoid carcinoma, as reported in a case report^24^. In addition, the infarcted tumor can sometimes clinically mimic malignancies because the tumor might cause more symptoms, e.g., pain, than an ordinary WT. For example, Eveson et al. reported pain as a symptom in 25% of the infarcted WTs and 7% in the ordinary WTs, in a study population of 323 WTs^25^. These particular findings can thus be possible pitfalls in diagnosing the tumor. In our infarcted case, the patient had experienced pain at some point. However, in the present patient series, as many as 25% of all patients had suffered from pain, which is more than has been reported by others^25^.

Previous studies have usually evaluated data mainly from a histological point of view, i.e., from histologically verified WTs. In our series, the majority (65%) of the WT patients underwent no surgery and showed no major concern regarding their WTs during follow-up. Only two patients (2%) who were initially followed up were later treated surgically, after 1.5–2 years, as both had increasing tumor growth and pain. It is also noteworthy that, in the surgically treated group, ten patients had initially had follow-up before the decision for surgery. Data on follow-up of WTs are scarce in the literature. In a study by Fíková et al., the focus was similar to ours in a series of 323 WT patients, although with a lower percentage of 34% of WTs that were treated conservatively, with a mean follow up time of 44.7 months. However, compared with our results, their series revealed more patients (16.5%) requiring surgery among those 109 patients with an initial follow-up decision, and two surgical specimens revealed malignancies (both adenocarcinomas). It is not clear, however, how reliable the preoperative FNAC statement was in the two malignant cases^26^.

Ten percent of the patients (n = 13/125) in our entire cohort had had surgery before 2016 because of an earlier WT and thus had a recurrent disease. These patients were included in the study series due to our initial study design and this was not known before the data collection was finalized. Naturally, the pathology report on FNAC might have been biased by this earlier diagnosis. The recurrent nature of WT indicates that operative treatment might not always be the best solution, as WTs can recur after surgery. Watchful waiting might be a better choice, especially with an asymptomatic patient, provided that the FNAC finding is certain enough for the diagnosis. Hence, further investigations of the long-term outcome of WT patients under conservative surveillance are important, as well as examining other less invasive treatment possibilities than surgery.

## Conclusion

The present series demonstrates that FNAC results indicating WTs were highly reliable when diagnostics was centralized to head and neck pathologists. Hence, it seems safe to manage an asymptomatic WT conservatively if the clinical findings support the benign nature of the tumor and the cytological statement indicates WT. However, the clinicians must be aware of the existing, although low, risk of diagnostic error of FNAC.

## Data Availability

The study data are available from the corresponding author.
